# Immunoglobulin λ Gene Rearrangement Can Precede κ Gene Rearrangement

**DOI:** 10.1155/1990/56014

**Published:** 1990

**Authors:** Jörg Berg, Mindy Mcdowell, Hans-Martin Jäck, Matthias Wabl

**Affiliations:** Department of Microbiology and Immulology University of California San Francisco California 94143-0414 USA

## Abstract

Immunoglobulin genes are generated during differentiation of B lymphocytes by joining gene segments. A mouse pre-B cell contains a functional immunoglobulin heavy-chain gene, but no light-chain gene. Although there is only one heavy-chain locus, there are two lightchain loci: κ and λ.It has been reported that κ loci in the germ-line configuration are never (in man) or very rarely (in the mouse) present in cells with functionally rearranged λ-chain genes. Two explanations have been proposed to explain this: (a) the ordered rearrangement theory, which postulates that light-chain gene rearrangement in the pre-B cell is first attempted at the κ locus, and that only upon failure to produce a functional κ chain is there an attempt to rearrange the λ locus; and (b) the stochastic theory, which postulates that rearrangement at the λ locus proceeds at a rate that is intrinsically much slower than that at the κ locus. We show here that λ-chain genes are generated whether or not the κ locus has lost its germ-line arrangement, a result that is compatible only with the stochastic theory.


*Developmental Immunology*, 1990, Vol. 1, pp. 53–57
Reprints available directly from the publisher
Photocopying permitted by license only

© 1990 Harwood Academic Publishers GmbH
Printed in the United Kingdom

### Immunoglobulin λ Gene Rearrangement Can Precede κ Gene Rearrangement

JÖRG BERG, MINDY MCDOWELL, HANS-MARTIN JÄCK and MATTHIAS WABL*

*Department of Microbiology and Immunology, University of California, San Francisco, California 94143-0414*

Immunoglobulin genes are generated during differentiation of B lymphocytes by joining gene segments. A mouse pre-B cell contains a functional immunoglobulin heavy-chain gene, but no light-chain gene. Although there is only one heavy-chain locus, there are two light-chain loci: κ and λ. It has been reported that κ loci in the germ-line configuration are never (in man) or very rarely (in the mouse) present in cells with functionally rearranged λ-chain genes. Two explanations have been proposed to explain this: (a) the ordered rearrangement theory, which postulates that light-chain gene rearrangement in the pre-B cell is first attempted at the κ locus, and that only upon failure to produce a functional κ chain is there an attempt to rearrange the λ locus; and (b) the stochastic theory, which postulates that rearrangement at the λ locus proceeds at a rate that is intrinsically much slower than that at the κ locus. We show here that λ-chain genes are generated whether or not the κ locus has lost its germ-line arrangement, a result that is compatible only with the stochastic theory.

KEYWORDS: Isotypic exclusion, κ germ-line arrangement, stochastic theory, ordered rearrangement theory.

### INTRODUCTION

Immunoglobulins consist of two identical heavy (H) chains and two identical light (L) chains. Both H and L chains consist of variable (V) and constant (C) regions. A given lymphocyte produces only one type of V region pair, i.e., it is monospecific. Although B lymphocytes are diploid, the products of only one allele encoding H, and one allele encoding L, are expressed on the cell membrane. This phenomenon, called allelic exclusion, is necessary to ensure the monospecificity of B lymphocytes (Pernis et al., 1965; Weiler, 1965). At the L-chain loci, monospecificity requires isotypic exclusion in addition to allelic exclusion. That is, a B cell expresses either κ or λ, but not both. In the mouse, the one Vλ1 gene segment (see Fig. 1) can combine with either the joining (J) λ1 or Jλ3 segment to form a λ1 or λ3 gene with the nearest C segment, and the one Vλ2 gene segment can join either to Jλ2 to form a λ2 gene or to Jλ1 or Jλ3 to form the rare genes that contain Vλ2 and Cλ1, or Vλ2 and Cλ3 (Blomberg et al., 1981; Reilly et al., 1984; Weiss et al., 1985). Isotypic exclusion extends also to these various genes within the λ locus.

In mouse κ-producing cells, the λ locus is rarely rearranged, but most of the λ-producing cells examined so far contained no κ loci in the germ-line configuration; only one κ locus in the germ-line configuration was detected in 11 λ-producing

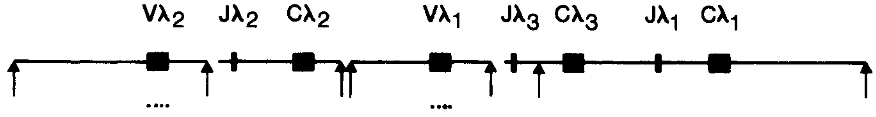

FIGURE 1. Schematic of the mouse λ locus depicting the *Eco*RI restriction fragments relevant for this study in the correct order and approximate length. Interruption of the line represents additional intervening sequences. The arrows denote *Eco*RI restriction sites. The dotted lines denote Vλ1 probe, which also hybridizes to Vλ2.

*Corresponding author.

hybridomas (Coleclough et al., 1981). In the mouse, there are over 200 V$\kappa$ genes, but only two V$\lambda$ genes. It was argued that the enzyme(s) joining the V gene segment to the J gene segment will have a greater chance of binding to a recognition sequence belonging to a V$\kappa$ than to a V$\lambda$ gene, and that the asymmetry in the behavior of the two L-chain loci could be explained by a simple stochastic model with unequal rate constants (Coleclough et al., 1981). The $\kappa$:$\lambda$ ratio of 95:5 in mouse serum is consistent with this view. However, in human serum, the $\kappa$:$\lambda$ ratio is 60:40, and in the human $\lambda$-producing B cells examined so far, there were no $\kappa$ loci in the germ-line configuration (Hieter et al., 1981). And, except for one case (Denny et al., 1985), there were no $\lambda$ rearrangements in human $\kappa$-producing cells (Hieter et al., 1981). This led to the idea, now widely accepted, that $\kappa$ rearrangement obligatorily precedes $\lambda$ rearrangement, and that $\lambda$ rearrangement will be attempted only if $\kappa$ rearrangement fails to produce a functional $\kappa$ gene (Hieter et al., 1981; Alt et al., 1986). On this theory, isotypic exclusion has a different basis than allelic exclusion.

To get a better understanding of what is happening at the L loci, it is important to consider the effect of $\kappa$ deletion. In the mouse, of the 22 $\kappa$ alleles in 11 $\lambda$-producing hybridomas investigated so far, 14 were nonproductively rearranged, 1 was in the germ-line configuration, and 7 were deleted (Coleclough et al., 1981). In human $\lambda$-producing cells, 1 of 20 $\kappa$ alleles was nonproductively rearranged, and 19 were deleted (Hieter et al., 1981). Thus, it is possible that the $\kappa$ alleles are deleted *after* $\lambda$ gene rearrangement, and that, therefore, the stochastic theory may have been prematurely dismissed.

### RESULTS AND DISCUSSION

### $\kappa$ Germ-Line Arrangement in a $\lambda$-Producing Myeloma

The first clue that the $\kappa$ alleles are deleted *after* $\lambda$ gene rearrangement came from our investigation of the $\lambda$-producing mouse myeloma J558, which has been reported to lack $\kappa$ loci in the germ-line configuration (Coleclough et al., 1981). On a Southern blot hybridized with a probe spanning all five of the J$\kappa$ regions (Hozumi et al., 1981), our J558 line shows the 3.7-kilobase $\kappa$ germ-line band of a *Xba*I digest

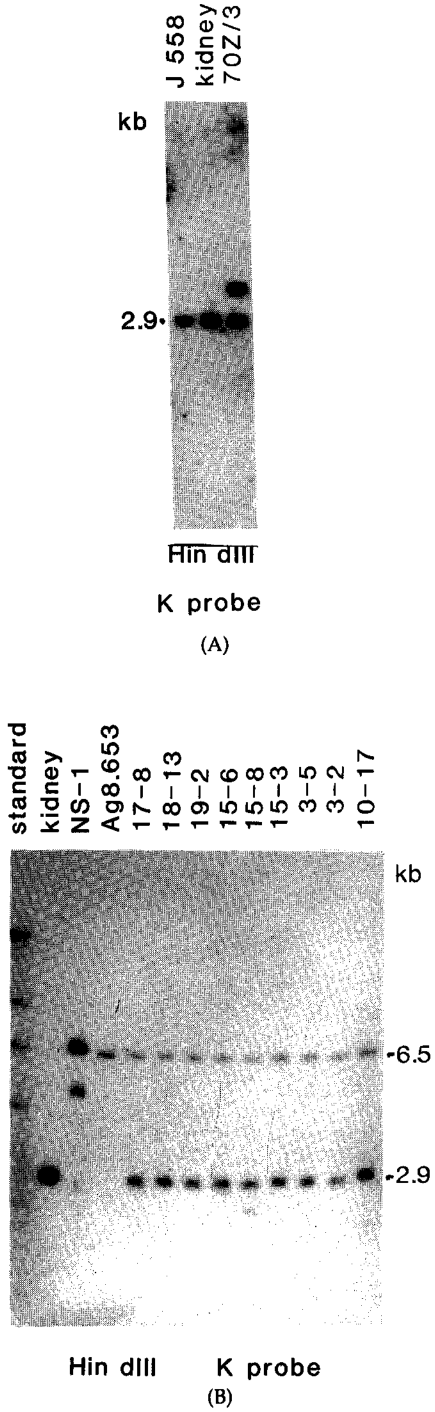

FIGURE 2. Southern blot analysis of *Hin*dIII-digested DNA from various mouse cell lines. The hybridizing $\kappa$ probe is a *Hin*dIII fragment containing all J$\kappa$ sequences (Hozumi et al., 1981). (A) J558, a $\lambda$1-producing myeloma; kidney, DNA representing germ-line configuration; 70Z/3, a $\kappa$-producing pre-B cell line. (B) Various $\lambda$-producing hybridomas between the myeloma Ag8.653 and lymphoblasts from spleen. NS-1, the parent of Ag8.653, synthesizes the $\kappa$ chain. Ag8.653 synthesizes no L chain. Molecular sizes in kb are indicated.

(not shown) and the 2.9-kb κ germ-line band of a *Hin*dIII digest (Fig. 2A). There are no bands indicative of nonproductive κ rearrangements. (The intensity of the 2.9-kb band is about half that of the kidney DNA band, because only half as much J558 DNA as kidney DNA was applied.) On a Southern blot that shows the rearrangement at the λ locus (Fig. 3), only a very faint band of the 3.5-kb germ-line Vλ1 gene segment is present. Because in the J558 line both Vλ1 gene segments are rearranged, this band represents contamination from connective tissue cells stemming from the tumor propagation in the mouse. In contrast, the germ-line band at the κ locus is as strong as in kidney DNA (Fig. 2A) and, therefore, cannot represent connective-tissue contamination alone. The same blot rehybridized with a probe spanning J3 and J4 of the H chain locus revealed a 7.0-kb band and a 4.4-kb band (Fig. 4). The bands representing rearrangements at the λ- and H-chain loci are identical to those reported by

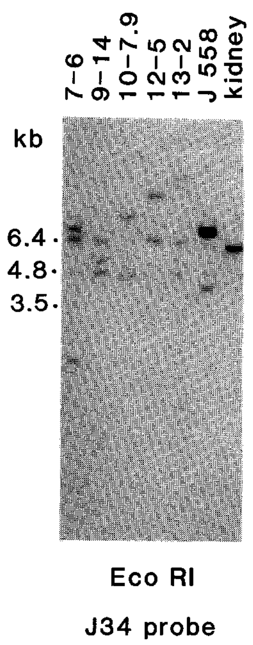

FIGURE 4. Southern blot analysis of *Eco*RI-digested DNA. The blot of Fig. 3 was rehybridized with a *Bam*HI-*Eco*RI fragment containing J3 and J4 (J34 probe). The 6.4-kb band is contributed by the fusion partner Ag8.653. Faint bands of 3.5 and 4.8 kb are from λ hybridization, as shown in Fig. 3.

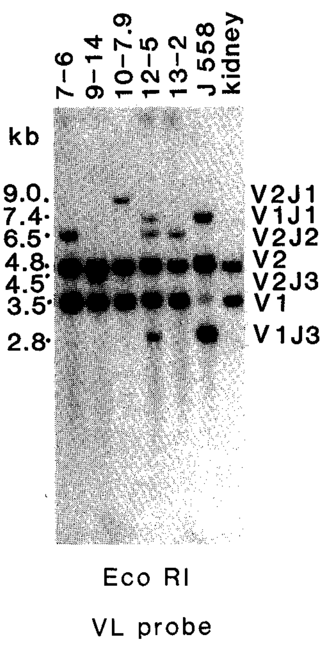

FIGURE 3. Southern blot analysis of *Eco*RI-digested DNA from various λ-producing mouse cell lines. The hybridizing Vλ (VL) probe is a 900-bp *Xba*I-*Hin*dIII fragment containing Vλ1. Because there is only one Vλ1, one Jλ1, and one Jλ3, the sizes of all possible *Eco*RI fragments hybridizing with a Vλ1 probe are predictable. The rearranged λ1 gene is contained in a 7.4-kb fragment and the λ3 gene in a 2.8-kb fragment; the germ-line Vλ1 fragment is 3.5 kb. Because the Vλ1 probe cross-hybridizes with Vλ2, it also detects all possible arrangements containing Vλ2: the germ-line band of Vλ2 is 4.8 kb, Vλ2 Jλ2 is 6.5 kb, Vλ2 Jλ1 is 9.0 kb, and Vλ2 Jλ3 is 4.5 kb. Because of the germ-line contribution of fusion parent Ag8.653, the intensities of the unrearranged λ bands are greater than those of the rearranged λ band.

Coleclough et al. (1981). Therefore, there is no doubt that their J558 line and our J558 line both stem from the same original transformed plasma cell. Because the κ germ-line band is retained in our J558 line, the κ locus in the germ-line configuration in their J558 line must have been lost *after* λ gene rearrangement.

### κ Germ-Line Arrangement in λ-Producing Hybridomas

If the κ germ-line sequences are easily lost in λ-producing cells, their presence might be detectable only in rather early B cells. Hybridomas derived from immunized mice or transformed human B cells might not be appropriate sources of λ-producing cells. We therefore investigated the λ-producing hybridomas derived from mitogen-stimulated B cells by Weiss et al. (1985). To obtain mostly λ-producing hybridomas, spleen cells from a κ-suppressed BALB/c mouse were used. The suppression of cells expressing κ should not change the mechanism of κ and λ gene formation. Treatment with antiserum to κ aborts the cells that express κ; pre-B cells that are about to rearrange the L-chain locus should not be affected by this unless κ-expressing cells influence, in some unknown way, the mechanism of λ-gene formation. The spleen cells were stimulated with lipopolysaccharide, and on day 3, the lymphoblasts were fused with the myeloma Ag8.653, and the resulting hybridomas were grown to $5 \times 10^8$ cells and frozen in liquid nitrogen. By the time DNA was isolated, the cells should not have divided more than 30 times, including the divisions of the original B cell. The hybridomas were typed according to their L-chain production by means of monoclonal antibodies to λ1 chain, and by $NaDodSO_4$/polyacrylamide gel electrophoresis of secreted proteins precipitated with a polyclonal antibody to λ chain. It was also possible to distinguish λ1 from λ2 and λ3 chains by differences in electrophoretic mobility. For the present study, λ-chain typing was confirmed by Southern blot analysis. An example is shown in Fig. 3.

Altogether, we examined 45 λ-producing hybridomas, ideally containing 90 κ alleles, derived from the mitogen-stimulated spleen B cells (Table 1). We detected 38 rearranged κ alleles. However, 19 hybridomas had at least 1 κ germ-line band in *Hin*dIII (Fig. 2B) and *Xba*I digests. The fusion partner Ag8.653 has no germ-line band, but has a 6.5-kb *Hin*dIII band, which is fainter than the germ-line band in the hybridomas. Of the 19 hybridomas with a κ germ-line band, 9 hybridomas showed no κ rearrangement, and 10 hybridomas showed a nonproductive rearrangement of one κ allele (Table 1).

TABLE 1

κ Alleles Contained in 45 λ-Producing Hybridomas[a]

| Number of hybridomas | $\kappa^0$ | $\kappa^-$ |
|---|---|---|
| 9 | + | 0 |
| 10 | + | 1 |
| 6 | 0 | 0 |
| 12 | 0 | 1 |
| 8 | 0 | 2 |

[a] $\kappa^0$, allele in germ-line configuration; +, at least one κ germ-line allele; 0, no κ germ-line allele. $\kappa^-$, nonproductively rearranged allele.

Some of the κ rearrangements might have gone undetected because of chromosomal loss in the hybridomas or because of the so-called RS rearrangement that would have deleted Jκ–Cκ (Durdik et al., 1984; Siminovitch et al., 1987). Because of the possibility that one allele has been deleted, we cannot determine whether hybridomas with no rearranged κ allele and a germ-line band have one or two alleles in the germ-line configuration. But we would like to point out that we have used the same κ probe that in previous work has detected only a few κ germ-line arrangements (Coleclough et al., 1981). The κ rearrangements differ in the various hybridomas. In two hybridomas, the 6.5-kb *Hin*dIII band of Ag8.653 is missing.

None of the hybridomas studied have more than two rearrangements at the H locus (the one from Ag8.653 is not counted) (Fig. 4). This confirms that they are clones and the product of the fusion of a single spleen cell with an Ag8.653 myeloma cell. The bands are different in different cells. Because H-gene rearrangement precedes L-gene rearrangement, this demonstrates that the λ-gene rearrangements in the fused spleen cells must represent independent events.

The fact that the κ locus may remain in its germ-line configuration in λ-producing cells excludes an obligatorily ordered progression in rearrangement activity from the κ locus to the λ locus. Our results do not rule out the possibility that only one κ allele must be rearranged in order for λ-gene formation to occur. However, this hypothesis would require some additional specification to explain isotypic exclusion. Furthermore, one would have to distinguish between the λ1, λ3, and λ2 isotypes. The simplest explanation of all the data is provided by the stochastic theory of Coleclough et al. (1981), which implies that the mechanism of isotypic exclusion is no different from the mechanism of allelic exclusion. For example, it has been suggested that an L chain, whether κ, λ1, λ2, or λ3, that allows expression of IgM on the cell surface (Alt et al., 1980) or that is able to displace BiP bound to a H chain (Wabl and Steinberg, 1982), shuts down further rearrangement activity at the immunoglobulin loci.

### MATERIALS AND METHODS

### Cell Lines

Cell lines were established and grown as described (Weiss et al., 1985; Jäck and Wabl, 1987).

### Southern Blot Analysis

Southern blot analysis was performed as described (Burrows et al., 1983). The plasmid with the 900-bp *Xba–Hin*dIII fragment containing Vλ1 was provided by H. Sakano, University of California, Berkeley; the plasmid with the *Hin*dIII fragment containing all Jκ sequences was provided by N. Hozumi, University of Toronto; and the plasmid with the *Bam*HI–*Eco*RI fragment containing J3 and J4 was provided by F. Blattner, University of Wisconsin, Madison.

### ACKNOWLEDGMENTS

We thank C. Steinberg for discussions, M. McKenney for editing the manuscript, and P. Winegar for typing it. We also thank H. Sakano, N. Hozumi, and F. Blattner for the DNA probes. This work was supported by National Institutes of Health grant 1 R01 GM37699-01A1, by a Norbert-Hoyer fellowship and a fellowship of the German Research Foundation to J.B., and by the Office of Health and Environmental Research, U.S. Department of Energy (contract no. DE-AC03-76-SF01012).

*(Received October 15, 1989)*

*(Accepted October 27, 1989)*

### REFERENCES

Alt F.W., Enea V., Bothwell A.L.M., and Baltimore D. (1980). Activity of multiple light chain genes in murine myeloma lines expressing a single, functional light chain. Cell **21:** 1–12.

Alt F.W., Blackwell T.K., DePinho R.A., Reth M.G., and Yancopoulos G.D. (1986). Regulation of genome rearrangement events during lymphocyte differentiation. Immunol. Rev. **89:** 5–30.

Blomberg B., Traunecker A., Eisen H., and Tonegawa S. (1981). Organization of four mouse $\lambda$ light chain immunoglobulin genes. Proc. Natl. Acad. Sci. USA **78:** 3765–3769.

Burrows P.D., Beck-Engeser G.B., and Wabl M.R. (1983). Immunoglobulin heavy-chain class switching in a pre-B cell line is accompanied by DNA rearrangement. Nature **306:** 243–246.

Coleclough C., Perry R.P., Karjalainen K., and Weigert M. (1981). Aberrant rearrangements contribute significantly to the allelic exclusion of immunoglobulin gene expression. Nature **290:** 372–378.

Denny C.T., Hollis G.F., Magrath I.T., and Kirsch I.R. (1985). Burkitt lymphoma cell line carrying a variant translocation creates a new DNA at the breakpoint and violates the hierarchy of immunoglobulin gene rearrangement. Mol. Cell. Biol. **5:** 3199–3207.

Durdik J., Moore M.W., and Selsing E. (1984). Novel $\kappa$ light-chain gene rearrangements in mouse $\lambda$ light chain-producing B lymphocytes. Nature **307:** 749–752.

Hieter P.A., Korsmeyer S.J., Waldmann T.A., and Leder P. (1981). Human immunoglobulin $\kappa$ light-chain genes are deleted or rearranged in $\lambda$-producing B cells. Nature **290:** 368–372.

Hozumi N., Hawley R.G., and Murialdo H. (1981). Molecular cloning of an immunoglobulin kappa constant gene from NZB mouse. Gene **13:** 163–172.

Jäck H.-M., and Wabl M. (1987). High rates of deletions in the constant region segment of the immunoglobulin $\mu$ gene. Proc. Natl. Acad. Sci. USA **84:** 4934–4938.

Pernis B., Chiappino G., Kelus A.S., and Gell P.G.H. (1965). Cellular localization of immunoglobulins with different allotypic specificities in rabbit lymphoid tissues. J. Exp. Med. **122:** 853–875.

Reilly E.B., Blomberg B., Imanishi-Kari T., Tonegawa S., and Eisen H.N. (1984). Restricted association of V and J-C gene segments for mouse $\lambda$ light chains. Proc. Natl. Acad. Sci. USA **81:** 2484–2488.

Siminovitch K.A., Moore M.W., Durdik J., and Selsing E. (1987). The human kappa deleting element and the mouse recombining segment share DNA sequence homology. Nucleic Acids Res. **15:** 2699–2705.

Wabl M., and Steinberg C. (1982). A theory of allelic and isotypic exclusion for immunoglobulin genes. Proc. Natl. Acad. Sci. USA **79:** 6976–6978.

Weiler E. (1965). Differential activity of allelic $\gamma$-globulin genes in antibody-producing cells. Proc. Natl. Acad. Sci. USA **54:** 1765–1772.

Weiss S., Meyer J., and Wabl. M.R. (1985). V$\lambda$2 rearranges with all functional J$\lambda$ segments in the mouse. Eur. J. Immunol. **15:** 765–768.

